# LARS is Associated with Lower Anastomoses, but not with the Transanal Approach in Patients Undergoing Rectal Cancer Resection

**DOI:** 10.1007/s00268-020-05876-6

**Published:** 2020-12-10

**Authors:** Alexandra Filips, Tobias Haltmeier, Andreas Kohler, Daniel Candinas, Lukas Brügger, Peter Studer

**Affiliations:** 1 Department of Visceral Surgery and Medicine, Inselspital, Bern University Hospital, University of Bern, Freiburgstrasse, 3000 Bern, Switzerland; 2 Department of Surgery, Hirslanden Klinik Beau-Site, Bern, Switzerland

## Abstract

**Background:**

Low anterior resection syndrome (LARS) is a defecation disorder that frequently occurs after a low anterior resection (LAR) with a total mesorectal excision (TME). The transanal (ta) TME for low rectal pathologies could potentially overcome some of the difficulties encountered with the abdominal approach in a narrow pelvis. However, the impact of the transanal approach on functional outcomes remains unknown. Here, we investigated the effect of the taTME approach on functional outcomes by comparing LARS scores between the LAR and taTME approaches in patients with colorectal cancer.

**Methods:**

We conducted a retrospective cohort study including 80 patients (*n* = 40 LAR-TME, *n* = 40 taTME) with rectal adenocarcinoma. We reviewed medical charts to obtain LARS scores 6 months after the rectal resection or a reversal of the protective ileostomy.

**Results:**

At the 6-month follow-up, 80% of patients exhibited LARS symptoms (44% minor LARS and 36% major LARS). LARS scores were not significantly associated with the *T*-stage, N-stage, or neo-adjuvant radiotherapy. The mean distance of the anastomosis from the anal verge was 4.0 ± 2.0 cm. The taTME group had significantly lower anastomoses compared with the LAR-TME group (median 4.0 cm [IQR1.8] vs. median 5.0 cm [IQR 2.0], *p* < 0.001). Univariable analysis revealed significantly higher LARS scores in the taTME group compared with the LAR-TME group (median LARS scores: 29 vs. 25, *p* = 0.040). However, multivariable regression analysis, adjusting for neo-adjuvant treatment, anastomosis distance from the anal verge, anastomotic leak rate, and body mass index, revealed no significant effect of taTME on the LARS score (adjusted regression coefficient:  − 2.147, 95%CI:  − 2.130 to 6.169, *p* = 0.359). We also found a significant correlation between LARS scores and the distance of the anastomosis from the anal verge (regression coefficient:  − 1.145, 95%CI:  − 2.149 to  − 1.141, *p* = 0.026).

**Conclusion:**

Fifty percentage of patients in this cohort exhibited some LARS symptoms after a mid- or low-rectal cancer resection. As previously described, LARS scores were negatively correlated with the distance of the anastomosis from the anal verge. TaTME was after adjustment for the height of the anastomosis not associated with higher LARS at 6 months when compared with LAR-TME.

## Introduction

In the past few decades, surgical treatment has significantly changed for patients with rectal cancer. Generally, the outcome for patients with rectal cancer [1] has significantly improved, due to the adoption of the total mesorectal excision (TME) [2] as a standard of care and the progress made in neo-adjuvant and adjuvant therapy. Currently, the open technique has increasingly been replaced with minimally invasive techniques. The benefits of minimally invasive surgery include a shorter hospital stay, fewer surgical site infections, and less postoperative pain. Nevertheless, studies have failed to show superiority for the minimally invasive approach, based on histopathological outcomes [3, 4]. Additionally, minimally invasive techniques cannot always overcome the technical difficulties of an oncological resection in a narrow male pelvis or the resection of a bulky malignancy in the mid-to-lower rectum. In these cases, the dissection of the mesorectal fascia sometimes cannot be completed down to the muscular pelvic floor. Recently, transanal (ta) TME evolved as a promising new technical variant to circumvent the problems associated with anterior rectal cancer resections in the lower pelvis [5]. This ‘bottom up’ approach potentially provides a better view for the dissection of the mesorectal fascia in the lower pelvis and allows a safe oncologically correct resection [6]. However, we lack studies that investigated the impact of the bottom-up approach on functional outcomes. As a complication after a rectal cancer resection, up to 80% of patients experience a complex of defecation problems, known as low anterior resection syndrome (LARS), and up to 50% experience severe defecation problems (major LARS) with impaired quality of life [7–9]. Therefore, the present study investigated the effect of the taTME approach on functional outcomes by comparing LARS scores between the LAR-TME and taTME treatments for patients with colorectal cancer. We hypothesized that the surgical technique, i.e., taTME vs. LAR-TME, does not affect LARS scores.

## Materials and methods

### Study design and patient inclusion

A retrospective cohort study was performed including 80 patients undergoing rectal cancer resection at the Department of Visceral Surgery and Medicine in the University Hospital of Bern, Switzerland from 2012 to 2017. This study meets the criteria of the STROBE guidelines (www.strobe-statement.org). LARS scores were routinely recorded in our outpatient clinic for all patients that underwent a resection of the rectum. For this study, we retrospectively retrieved data from electronic patient charts. We analyzed LARS scores of patients treated for rectal adenocarcinoma with the LAR-TME (laparoscopic or open) or the taTME approach in our tertiary center. We adopted taTME in our institution after formal training in 2015. Therefore, the LAR-TME group included a series of consecutively treated patients with the traditional LAR approach between 2012 and 2015, and the taTME group correspondingly included the first patients treated with the transanal approach between 2015 and 2017.

We analyzed the LARS scores recorded six months after closure of the protective loop ileostomy or six months after resection of the rectum, in patients without a diverting ileostomy. We used the LARS score validated by Emmertsen et al. [10] and the following proposed categories: 0–20 points indicated ‘No LARS,’ 21–29 points indicated ‘Minor LARS,’ and 30–42 points indicated ‘Major LARS.’ All procedures were performed by two fully trained colorectal surgeons.

All patients were preoperatively evaluated at the multidisciplinary team (MDT) meeting for colorectal cancer. Patients received surgery alone or neo-adjuvant treatment, according to the S3 guidelines (https://www.awmf.org/leitlinien/detail/ll/021-007OL.html) and the decision made at the MDT meeting.

## Perioperative management

All patients underwent a mechanical bowel preparation the day before surgery and received prophylactic intravenous antibiotics before the skin incision.

LAR-TME: A medial-to-lateral approach was used in laparoscopic treatments and a lateral-to-medial approach was used for open surgery, as described elsewhere [11]. A high-tie of the inferior mesenteric artery was performed. The superior mesenteric vein was dissected at the inferior border of the pancreas. The splenic flexure was mobilized in all patients.

TaTME: A Gelpoint (Applied Medical, California, USA) and Airseal system (Conmed, New York, USA) was used as previously described [12]. All taTMEs were performed in a two-team procedure. The specimen was retrieved through an abdominal incision (Pfannenstiel or periumbilical mini-laparotomy). The anastomosis of the descending colon to the rectum was performed end–end or side–end with a circular stapler. For very low malignancies, we fashioned a hand-sewn end-end anastomosis (colo-anal pull through). 88% of patients received a protective diverting loop ileostomy. The postoperative period was guided by the enhanced recovery after surgery guidelines [13].

We obtained approval from the local Ethics Board (Ethics committee of the Canton of Berne Switzerland, registration number: 2018–01,911) before study initiation.

## Statistical analysis

Missing data were found in two variables (TNM lymph node stage and time to stoma reversal) and reported in the baseline characteristics (Table 1). The normality of distribution of continuous variables was assessed using histograms, skewness, and the Shapiro–Wilk test. Categorical variables were compared with Fisher’s exact test or Pearson’s chi-square test. Continuous variables were analyzed with the Mann–Whitney *U* test. Results were reported as the number and percentage or the median and interquartile range (IQR), as appropriate. *P* values < 0.05 were considered statistically significant.

**Table 1:** Baseline characteristics

Patient characteristics	All	LAR-TME	taTME	*p* value^†^
	(*n* = 80)	(*n* = 40)	(*n* = 40)	
Age (*y*)*	62.0 (25.0)	60.5 (23.0)	62.0 (29.0)	0.862^#^
BMI (kg/m^2^)*	25.0 (5.3)	24.3 (6.4)	25.3 (5.0)	0.583^#^
Male sex	53 (66.3)	26 (65.0)	27 (67.5)	1.000
ASA Classification				
1	8 (10.0)	2 (5.0)	6 (15.0)	0.215^‡^
2	24 (30.0)	11 (27.5)	13 (32.5)	
3	42 (52.5)	25 (62.5)	17 (42.5)	
4	6 (7.5)	2 (5.0)	4 (10.0)	
Neo-adjuvant treatment	44 (55)	20 (50.0)	24 (60.0)	0.500
NRS ≥ 3	39 (48.8)	20 (50.0)	19 (47.5)	1.000
Tumor characteristics				
Tumor distance from AV (cm)*	8.0 (5.0)	9.0 (4.0)	7.0 (5.0)	0.023^#^
TNM T-stage				
0	5 (6.3)	3 (7.5)	2 (5.0)	0.302^‡^
1	19 (23.8)	8 (20.0)	11 (27.5)	
2	21 (26.3)	11 (27.5)	10 (25.0)	
3	28 (35.0)	12 (30.0)	16 (40.0)	
4	7 (7.8)	6 (15.0)	1 (2.5)	
TNM N-stage positive	27/79 (33.8)	11/39 (28.2)	16/40 (40.0)	0.344
Surgery-related data		†		
Anastomosis distance from AV (cm)*	4.0 (2.0)	5.0 (2.0)	4.0 (1.8)	< 0.001^#^
Operative time (min)*	300 (100)	320 (98)	275 (98)	0.010^#^
Blood loss (ml)*	300 (300)	400 (288)	200 (238)	0.014^#^
Stapled anastomosis	68 (85)	38 (95)	30 (75)	0.025
Loop ileostomy	71 (89)	36 (90.0)	35 (87.5)	1.000
*Mercury*				
1	72 (90.0)	35 (87.5)	37 (92.5)	0.549^‡^
2	7 (8.8)	4 (10.0)	3 (7.5)	
3	1 (1.3)	1 (2.5)	0 (0.0)	
CRM positive	3 (3.8%)	3 (7.5)	0 (0.0)	0.241
Anastomotic leak	9 (11.3)	4 (10.0)	5 (12.5)	1.000
Time to stoma closure, *n* = 75 (days)*	118 (147)	123 (145)	118 (150)	0.803^#^

Values are numbers (percentages) of patients, unless indicated otherwise. *Values are medians (interquartile range). ^†^Fisher’s exact test, unless indicated otherwise. ^‡^Pearsons’s chi-square test. ^#^Mann–Whitney U test

*BMI* Body Mass Index, *ASA* American Society of Anesthesiologists, *NRS* nutritional risk score, *AV* anal verge, *CRM* circumferential resection margin

The effect of the taTME technique on the LARS score at 6 months postoperatively was analyzed in multivariable linear regression analysis adjusted for other clinically important variables. Clinically important variables were correlated with the LARS score at 6 months in univariable analysis. Variables with *p* values < 0.1 were entered in the multivariable regression model. Interactions between the taTME technique and other independent variables included in the regression model were assessed in separate regression analyses. The degree of multicollinearity between predictor variables was assessed using the variance inflation factor (VIF). A VIF < 5 was assumed to exclude significant collinearity. Results of the multivariable regression analysis were expressed as regression coefficient (RC) and 95% confidence interval (95%CI). Statistical analyses were performed with SPSS Statistics (IBM Corporation, Armonk, New York, USA).

## Results

### Patients and tumor characteristics

Among the 80 patients studied (*n* = 40 LAR-TME, *n* = 40 taTME), the majority were male (*n* = 53, 66%), median age was 62 years (IQR 25), and median BMI 25 kg/m^2^ (IQR 5.4). At the time of diagnosis, 39 patients (49%) presented with malnutrition (nutritional risk scores ≥ 3, [14]) and received nutritional support before the resection. Overall, 48 (58%) participants had significant comorbidities, defined as an American Society of Anesthesiologists score ≥ 3. Forty-four (55%) patients received neo-adjuvant treatment. Patient characteristics were not significantly different between the taTME and LAR-TME groups (Table 1).

We found a *T*-stage ≥ 3 in 35 (44%) patients and a positive *N*-stage in 27 (34%) patients, with no significant difference between the groups. Overall, the median distance from the anal verge (AV) to the distal end of the tumor was 8.0 cm (IQR 5 cm). The median tumor height from the AV was significantly lower in the taTME group compared with the LAR-TME group (7.0 cm [IQR 5.0] vs. 9.0 cm [IQR 4.0], *p* = 0.023; Table 1).

## Surgery-related data and histopathology

Overall, the median operative time was 300 min (IQR 100). The operative time was significantly shorter in the taTME compared with the LAR-TME group (320 min [IQR 98] vs. 275 min [IQR 98], *p* = 0.010). In addition, intraoperative blood loss was significantly lower in patients undergoing taTME compared with LAR-TME (200 ml [IQR 238] vs. 400 ml [IQR 288], *p* = 0.014). The median anastomotic height (distance from the AV to the anastomosis) was significantly lower in theTME compared with the LAR-TME group (4 cm [IQR1.8] vs. 5 cm [IQR 2.0], *p* < 0.001). A stapled anastomosis was performed in 68 (85%) patients. The remaining patients received a hand-sewn anastomosis. The hand-sewn anastomosis was performed significantly more frequently in the taTME vs. the LAR-TME group (*n* = 10 [25%] vs. *n* = 2 [5%], *p* = 0.025). Overall, 71 (88.8%) patients received a protective loop ileostomy. The surgical specimen (MERCURY 1) was of good quality in 35 (87.5%) patients in the LAR-TME group and in 37 (92.5%) patients in the taTME group (*p* = 0.549). A positive circumferential resection margin (CRM) was found in three (3.8%) patients in the LAR-TME group and no patients in the taTME group (*p* = 0.241).

**Fig. 1:**
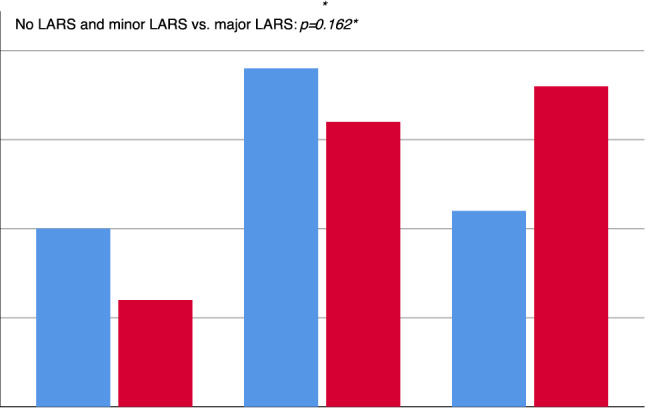
LARS scores after LAR TME vs. taTME 6 month after ileostomy closure. Number of patients with no LARS minor LARS and major LARS in the LAR-TME group (blue, *n* = 40) compared with the taTME group (red, *N* = 40). “Fisher’s exact test” LARS: Low anterior resection syndrome. LAR TME: Low anterior resection and total mesorectal excision. taTME Transanal total mesorectal excision.

Overall, protective loop ileostomies were closed at a median of 118 days (IQR 147) after rectal surgery. Median time to stoma closure was comparable between the LAR-TME and taTME group (123.5 days [IQR 145] vs. 118.0 days [IQR 150] *p* = 0.803). Moreover, the time to stoma closure after resection had no impact on the LARS scores at 6 months (Fig. 1). No significant association between anastomotic leakage and the time of stoma closure was found (median 131 [IQR 137] vs. 114 [IQR 139] days, *p* = 0.308), as well as in the taTME (median 131 [IQR 145] vs. 104 [IQR 140], *p* = 0.294) and LAR-TME (median 155 [IQR 182] vs. 123 [IQR 141] days, *p* = 0.716) group. We detected an anastomotic leak in nine (11.3%) patients. The leakage rates were not significantly different between groups (LAR-TME *n* = 4 [10%] vs. taTME *n* = 5 [12.5%]; *p* = 1.000). However, anastomotic leakage was associated with high LARS scores. The median LARS score was 34 (IQR 9) for patients with leakage and 26 (IQR 10) for patients without leakage (*p* = 0.259).

## LARS score

The LARS scores at six months after closure of the ileostomy or after resection of the rectum (in cases where no stoma was created) were evaluated (Fig. 1). The median overall LARS score was 27 (IQR 12). A total of 64 (80%) patients presented with LARS symptoms, including fecal incontinence or urgency, frequent or fragmented bowel movements, emptying difficulties, or increased intestinal gas. Of the 64 patients with LARS symptoms, 35 (44%) had minor LARS (44%) and 29 (36%) had major LARS. LARS symptoms were present in 30 (75%) patients in the LAR-TME group and 34 (85%) in the taTME group.

**Fig. 2:**
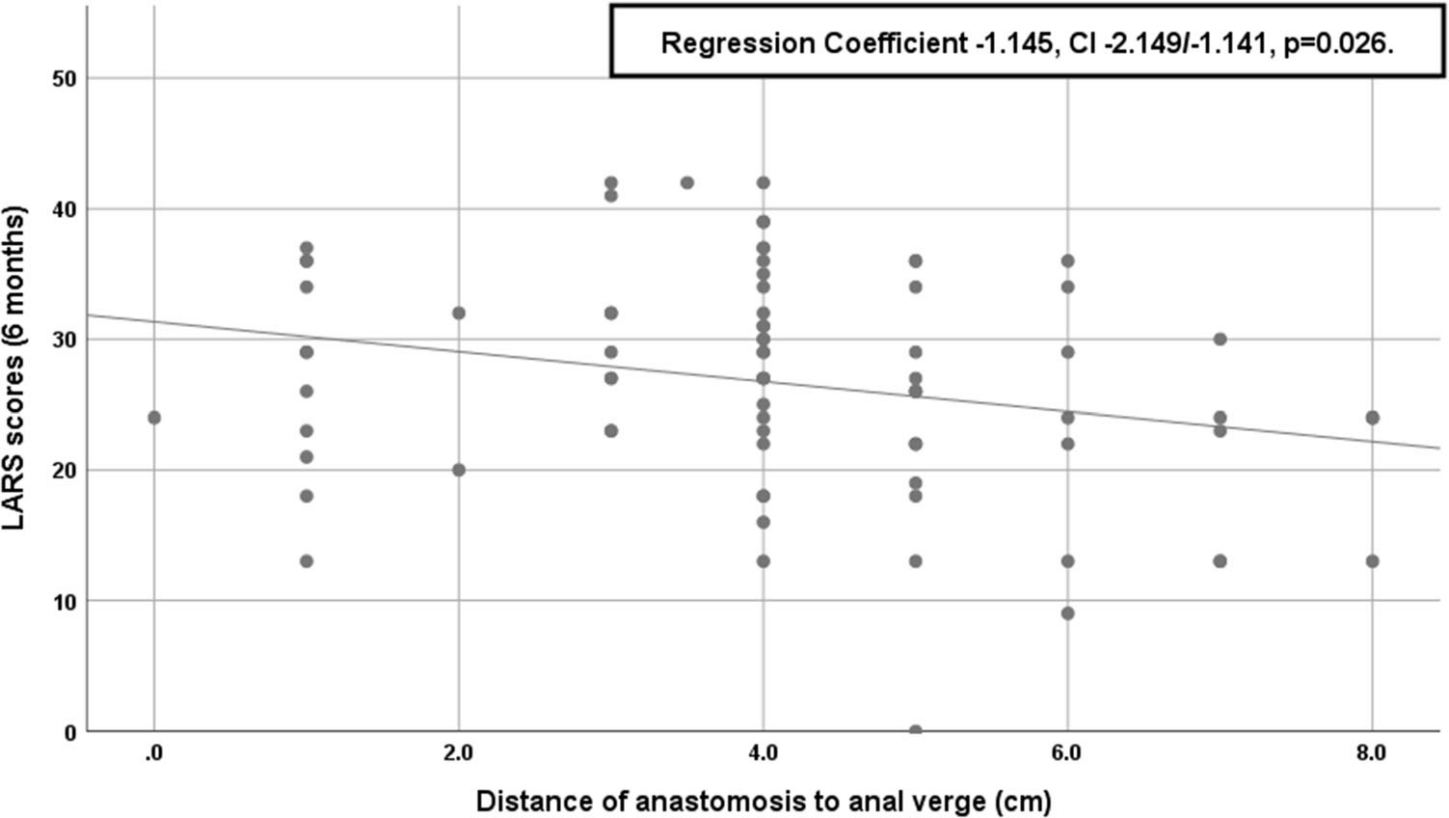
LARS scores correlate with the distance of the anastomosis from the anal verge. Six-month LARS scores decrease with increasing distance between the anastomosis and the anal verge at the 6 month time point. CI: Confidence interval. LARS: Low anterior resection syndrome

The proportions of no LARS, minor LARS, and major LARS were not significantly different between the LAR-TME and taTME groups (*p* = 0.249). Univariable analysis showed that median LARS scores were significantly higher in the taTME group compared with the LAR-TME group (29 [IQR 13] vs. 25 [IQR 11], *p* = 0.040). However, in multivariable regression analysis, after adjusting for neo-adjuvant treatment, anastomosis distance from the AV, the anastomotic leak rate, and BMI, the taTME approach was not significantly associated with LARS scores (adjusted regression coefficient (RC) 2.011, 95%CI  − 2.147 to 6.169, *p* = 0.338). The variables tested in the multivariable regression model showed no significant interactions or colinearity (VIF < 5 for all variables). Large distances between the anastomosis and the AV were associated with significantly lower LARS scores (regression coefficient  − 1.145, 95%CI:  − 2.149 to  − 1.141, p = 0.026; Fig. 2). The time to stoma closure (days) was associated with higher LARS scores, but this effect was not statistically significant (regression coefficient 0.013, 95%CI:  − 0.003 to 0.029, *p* = 0.114). No significant association between LARS and male sex compared with female sex was found (Table 2). LARS scores were not significantly higher in patients after an anastomotic leakage compared with patients without leakage (median 36 [IQR 18] vs. 27 [IQR 10], *p* = 0.259).

**Table 2:** Association between male/female sex and LARS

	No LARS	Minor LARS	Major LARS	*p* value^†^
	(*n* = 16)	(*n* = 35)	(*n* = 29)	
Male sex Female sex	10 (18.9)	24 (45.3)	19 (35.8)	0.953
	6 (22.2)	11 (40.7)	10 (37.0)	
	No LARS or minor LARS		Major LARS	
Male sex Female sex	34 (64.2) 17 (63.0) Median LARS		19 (35.8)	1.000
			10 (37.0)	
			IQR	
Male sex	26 29		13	0.939^#+^
Female sex			11	

Values are numbers (percentages) of patients, unless indicated otherwise

^†^Fisher’s exact test, unless indicated otherwise. ^#+^Mann–Whitney *U* test

*LARS* Low anterior resection syndrome, *IQR* interquartile range

To determine whether the learning curve might have affected the implementation of the taTME, we compared the tumor height and the distance of the anastomosis from the AV in our first 20 cases to those in the consecutive 20 cases of this cohort. We found no significant differences between the first and second 20 patients that underwent taTME in the median tumor height (7.0 cm [IQR 7.0] vs. 7.0 cm [IQR 4.0]; *p* = 0.838) or the median distance of the anastomosis from the AV (4.0 cm [IQR 3.0] vs. 4.0 cm [IQR 1.0]; *p* = 0.380). Finally, we have not observed any local recurrences at the time of the analysis of this cohort.

## Discussion

Some concern has arisen over the functional outcomes after taTME, due to the placement of a large port in the anal canal for a relatively long time period. In this study, we evaluated the LARS score as a surrogate marker for ano-rectal function after TME [15] [16]. We compared outcomes between our first consecutive 40 taTME operations and 40 LAR-TME operations performed prior to the introduction of taTME in our institution. We found that the taTME approach did not negatively impact the LARS scores.

The groups had similar overall patient characteristics and rectal tumor characteristics, except for the tumor distance from the AV. We observed significantly lower tumors in the taTME group than in the LAR-TME group. This finding can be explained by the fact that the taTME technique was preferred for treating lower tumors, and the LAR-TME was preferred over the taTME for treating high rectal tumors, particularly in female patients, in our institution. Accordingly, we found significantly lower anastomoses in the taTME compared with the LAR-TME group. Consistent with this result, the hand-sewn pull-through type of anastomosis was used significantly more frequently in the taTME group than in the LAR-TME group.

We found that the operative time was significantly shorter in patients undergoing taTME compared with LAR-TME. This result can be explained by the fact that the taTME was always performed with a two-team approach. Additionally, we observed significantly less blood loss with the taTME vs. the LAR-TME approach. We hypothesize that this finding reflects the improved view of the dissection plane, and, therefore, the greater safety in lower pelvis dissections performed with the taTME compared with the LAR-TME approach. A pathological examination of the surgical specimens showed no differences between the groups in terms of the Mercury grade or the CRM positivity. However, we observed a tendency toward more Grade 1 Mercury specimens and less CRM positive specimens in the taTME group compared with the LAR-TME group, as reported previously [17].

As described in several previous studies, we confirmed that a high percentage of patients showed LARS symptoms after the oncological resection of the rectum [9]. In our cohort, 80% of patients presented with LARS symptoms, and of those, 36% had severe major LARS. However, a detailed analysis showed that the incidences of minor and major LARS were not different between the taTME and LAR-TME groups (Fig. 1). Univariate analysis showed a higher major LARS rate after the taTME compared with the LAR-TME. This can be explained by the significantly lower level of anastomosis with the taTME (Fig. 2). Furthermore, we demonstrated a significant correlation between the anastomosis distance from the AV and the severity of LARS, which is generally accepted in the field. The other parameters studied in our cohort did not have a negative impact on LARS symptoms. In particular, the BMI, neo-adjuvant treatment, time to stoma closure, and anastomotic leakage had no statistical impact on LARS symptoms in either group.

In our opinion, the taTME has technical advantages over the LAR-TME approach for oncological resections of low rectal tumors that require a full TME. These advantages are most striking when the TME is performed in a difficult male pelvis or to remove bulky, low tumors (< 10 cm). In the case of very low rectal malignancies, the transanal approach might enable the possibility for a correct oncological resection with an anastomosis instead of an amputation. However, the taTME is a demanding surgical procedure with relevant pitfalls, and it requires standardized training [18]. Furthermore, as demonstrated in the current study, lower anastomoses are associated with a higher complication rate, including LARS. Consequently, very low colorectal anastomoses should be avoided whenever possible and oncologically safe, regardless of the technique used for rectal resections.

We believe that the taTME procedure should be in the armamentarium of colorectal surgeons in specialized units treating low rectal pathologies. The technique should be restricted to selected patients (mainly male patients with low pathologies, especially bulky malignancies). Compared with open and laparoscopic surgery, the robotic approach offers technical benefits in the low pelvis as well and is of course a contender in this type of surgery.

This study had some relevant limitations. The retrospective design had inherent limitations. The temporal separation of the cohorts might have resulted in a selection bias (significantly lower tumors in the taTME cohort). Furthermore, we obtained no quality of life questionnaires and focused solely on the LARS score to assess ano-rectal function after the rectal resection. However, several previous studies revealed a solid correlation between the LARS score and bowel-related quality of life [7]. Despite these limitations, this study adds valuable data regarding the functional outcome in patients undergoing taTME.

In conclusion, taTME was after adjustment for the height of the anastomosis not associated with higher LARS at 6 months when compared with LAR-TME. Further studies are warranted, especially to analyze the long-term oncological outcome.
